# The Association Between Alcohol Consumption and Left Ventricular Ejection Fraction: An Observational Study on a General Population

**DOI:** 10.1097/MD.0000000000003763

**Published:** 2016-05-27

**Authors:** Zhao Li, Xiaofan Guo, Yinglong Bai, Guozhe Sun, Yufan Guan, Yingxian Sun, Abraham Maria Roselle

**Affiliations:** From the Department of Cardiology (ZL, XG, GS, YS), the First Hospital of China Medical University; Department of Maternal and Child Health (YB), School of Public Health, China Medical University, Shenyang, Liaoning, China; and Department of Cardiology (YG, AMR), Johns Hopkins University, Baltimore, MD.

## Abstract

The results of previous studies on the relation between alcohol consumption and heart failure (HF) have been inconsistent. This study aimed to evaluate the association between alcohol consumption and left ventricular ejection fraction (LVEF) in a general population.

A total of 10,824 adults were examined using a multistage cluster sampling method to select a representative sample of individuals who were at least 35-years old. The participants were asked to provide information about their alcohol consumption. Echocardiograms were obtained, and LVEF was calculated using modified Simpson's rule.

Of the 10,824 participants included in the present study, 46.1% were males, and the mean participant age was 54 years; age ranged from 35 to 93 years. The overall prevalence of LVEF< 0.50 and LVEF < 0.40 in the studied population was 11.6% and 2.9%, respectively. The prevalence of LVEF < 0.5 and LVEF < 0.04 was higher in both the moderate and heavy drinker groups than in the nondrinker group (*P* <0.05). Multivariate logistic regression analyses corrected according to the different levels of alcohol consumption showed that moderate and heavy drinkers had an –1.3-fold and 1.2-fold higher risk of LVEF <0.5, respectively, than nondrinkers (OR: 1.381, 95% CI: 1.115–1.711, *P* = 0.003 for moderate drinkers; OR: 1.246, 95% CI: 1.064–1.460, *P* = 0.006 for heavy drinkers). Heavy drinkers had an ∼1.5-fold higher risk of decreased LVEF < 0.4 than nondrinkers (OR: 1.482, 95% CI: 1.117–1.965, *P* = 0.006). Moderate drinkers did not show a risk of decreased LVEF < 0.4 that was significantly higher than that of nondrinkers (OR: 1.183, 95% CI: 0.774–1.808, *P* = 0.437).

According to these results, we concluded that increased alcohol consumption was associated with decreased LVEF compared with no alcohol consumption in this general population.

## INTRODUCTION

The effect of alcohol on the heart is a controversial issue. Some studies have reported that alcohol intake can reduce the risk of coronary heart disease,^1,2^ whereas other studies have reported that alcohol use is associated with increased cardiac morbidity and mortality.^3–5^ The results of previous studies on alcohol consumption and heart failure (HF) have also been inconsistent. The Framingham Heart Study reported that alcohol consumption is not associated with an increased risk of congestive HF, even among heavy drinkers (>15 drinks/week in men and >8 drinks/week in women).^6^ The Olmsted County Heart Function Study concluded that in a community setting, light alcohol consumption (<1 drink a day) was associated with a reduced prevalence of moderate systolic dysfunction.^7^ The British Regional Heart Study concluded that there was no evidence that light to moderate drinking is beneficial for the prevention of HF in older men without a history of MI. However, heavier drinking (≥5 drinks/day) was associated with an increased risk of HF in susceptible men with underlying myocardial ischemia.^8^

The latest study indicates that HF has been an important cause of cardiac mortality globally, with increasing prevalence and incidence.^9–11^ Although left ventricular (LV) performance can be evaluated by multiple parameters, including LV dimensions, the LV mass index, regional wall motion abnormalities, diastolic dysfunction, and left ventricular ejection fraction (LVEF) are the most representative measures of LV systolic function.^12,13^ However, few studies have focused on the association between alcohol consumption and HF among the general population; clarifying this relationship will be useful for the management of individuals with HF from a clinical perspective. Thus, we performed this study based on a general population from rural China with the aim of examining the association between alcohol consumption and depressed LVEF.

## Methods

### Study Population

This study adopted a multistage, stratified, random cluster sampling scheme. From January 2012 to August 2013, a representative sample of individuals who were at 35 years or older was selected in order to examine the prevalence, incidence, and natural history of cardiovascular risk factors in Liaoning Province. This research was approved by the Ethics Committee of China Medical University (Shenyang, China). Procedures were performed in accordance with ethical standards. The detailed methods have been previously published.^41,44,45^ We used baseline data in this report, and only participants who had complete data sets for the variables analyzed were included, yielding a final sample size of 10,824 individuals (4989 males and 5835 females).

### Data Collection and Definitions

Via an interview with a standardized questionnaire, the data were obtained on demographics, lifestyle, dietary habits, income, history of heart disease, and medication used over the past 2 weeks .Blood samples were obtained from all the subjects in the morning after at least 12 hours of fasting. Serum potassium and magnesium, as well as other routine blood biochemical indexes, were analyzed using an autoanalyzer (Olympus AU 640; Olympus Corp., Kobe, Japan). The detailed process have been presented elsewhere.^14,46,47^

Hypertension was defined as SBP ≥140 mm Hg, DBP ≥90 mm Hg and/or use of antihypertensive medications according to the JNC-7 report.^15^ And according to the World Health Organization (WHO) criteria, body mass index (BMI) values were categorized into different groups^16^: normal, overweight, and obese (for normal, BMI <25 kg/m^2^; for overweight, 25≤ BMI <30 kg/m^2^; and for obese, BMI ≥30 kg/m^2^). Participants with waist circumference (WC) ≥88 cm for females and WC ≥102 cm for males were defined as abdominal obesity.^17^ Dyslipidemia was determined by the National Cholesterol Education Program-Third Adult Treatment Panel (ATP III) criteria.^18^ FPG ≥7 mmol/L (126 mg/dL) and/or receiving treatment for diabetes was diagnosed as diabetes mellitus according to the following WHO criteria.^19^ Using the Chronic Kidney Disease Epidemiology Collaboration (CKD-EPI) equation, the glomerular filtration rate (GFR) was estimated.^20,47^ Reduced GFR was defined as an estimated GFR (eGFR) <60 mL/min/1.73 m^2^. In accordance with WHO criteria, anemia was defined as serum hemoglobin levels <12.0 g/dL (<120 g/L) for women and <13.0 g/dL (<130 g/L) for men^21^ Uric acid levels >422 μmol/L for men and >363 μmol/L for women was defined as hyperuricemia.^22^

### Echocardiography Measurements

The transthoracic echocardiogram included M-mode, 2-dimensional, and color Doppler. Detailed measurements used in this study have been previously published.^41,48^ According to the guideline of American Society of Echocardiography,^39^ left ventricular internal dimensions (LVIDs), posterior wall thickness (PWT),as well as interventricular septal thickness (IVST) were obtained.^40^ Left ventricular volumes were obtained from the apical 4-chamber view and the left ventricular ejection fraction (LVEF) was computed using the modified Simpson's rule.^23,24^ We used thresholds of LVEF < 0.5 and LVEF < 0.4 to indicate decreased LVEF.^25,26^

### Alcohol Consumption Assessment

Alcohol consumption was assessed during the interview using the questionnaire. The participants were asked to provide information regarding whether they regularly consumed alcohol, the average amount of alcohol they consumed per day, and the number of days per month that they consumed alcohol. The amount of pure alcohol consumed was calculated based on the reported frequency and amount of drinking. In China, the ethanol weight content differed among the beverages as follows: 5% in beer, 12.5% in red wine, and 45% in hard liquor. One drink was defined as an average of 15 g of ethanol.^37^ We used thresholds based on the definition of daily alcohol consumption from the National Institute on Alcohol Abuse and Alcoholism to categorize the participants based on their level of consumption as follows: nondrinkers (i.e., abstainers, or no alcohol consumption history), moderate drinkers (i.e., up to 1 drink/day for women and up to 2 drinks/day for men), and heavy drinkers (i.e., >1 drink/day for women and >2 drinks/day for men).^38^

### Statistical Analysis

Descriptive statistics were calculated for all the variables; continuous variables are reported as the mean values and standard deviations, and categorical variables are reported as counts and percentages. Differences among the categories were evaluated using nonparametric tests or the χ2 test, as appropriate. Multivariate logistic regression analyses were used to identify correlates of decreased LVEF; the association strengths are expressed as odds ratios (ORs) and corresponding 95% confidence intervals (CIs). All the statistical analyses were performed using SPSS version 22.0 and *P* values <0.05 were considered statistically significant.

## RESULTS

### Basic Characteristics of the Study Population

Of the 10,824 participants included in the present study, 46.1% were males; the mean age was 54 years and ranged from 35 to 93 years. Of all the participants, 810 (7.5%) and 1820 (7.5%) were moderate and heavy drinkers, respectively. On average, the LVEF calculated using the modified Simpson's rule was 0.62 ± 0.10 among the included population. The overall prevalence of LVEF< 0.50 and LVEF < 0.40 in the included population was 11.6% and 2.9%, respectively. Table 1 shows the clinical characteristics and demographics of the study population according to the alcohol consumption level. The mean age of participants in the nondrinker, moderate, and heavy drinker groups was 53.75 ± 10.56, 54.20 ± 10.79, and 53.74 ± 10.30 years, respectively. No significant differences were found among the 3 groups in the demographic or and laboratory variables included in the table, except in FPG. The prevalence of comorbidities, history of stroke, history of heart disease, and diabetes was significant different among the 3 groups (all *P* < 0.001).

**TABLE 1:**
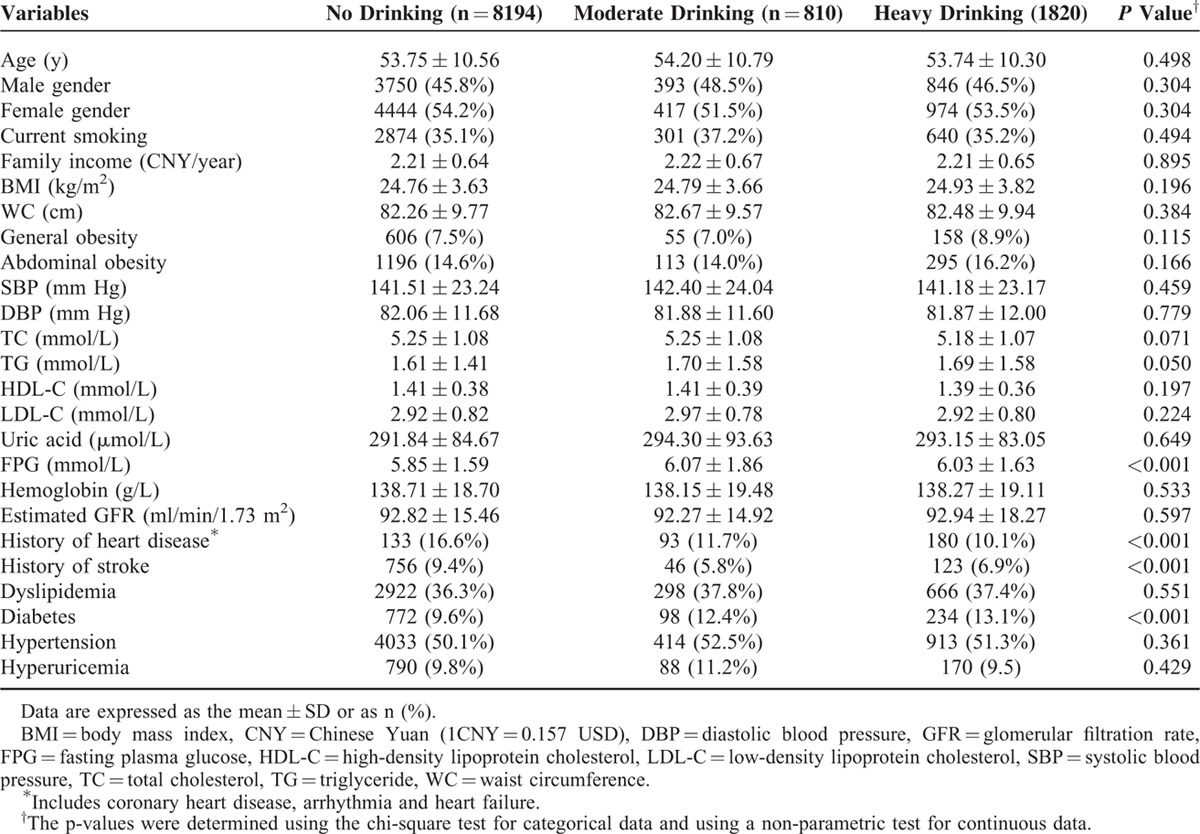
Baseline Characteristics of the Study Population According to Alcohol Consumption Groups (n = 10,824)

### Mean Level of LVEF and Prevalence of Decreased LVEF With Different Levels of Alcohol Consumption

**FIGURE 1:**
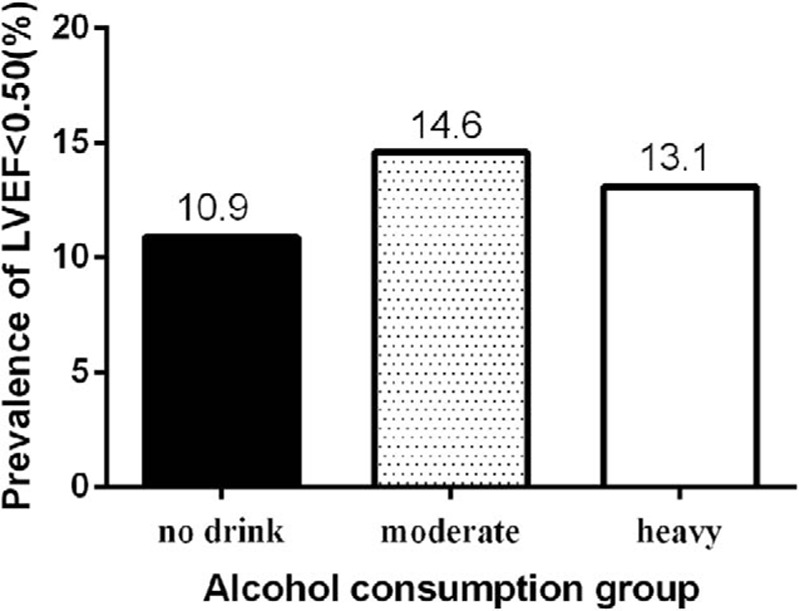
Prevalence of LVEF < 0.50 in the different alcohol consumption groups. LVEF = left ventricular ejection fraction.

**FIGURE 2:**
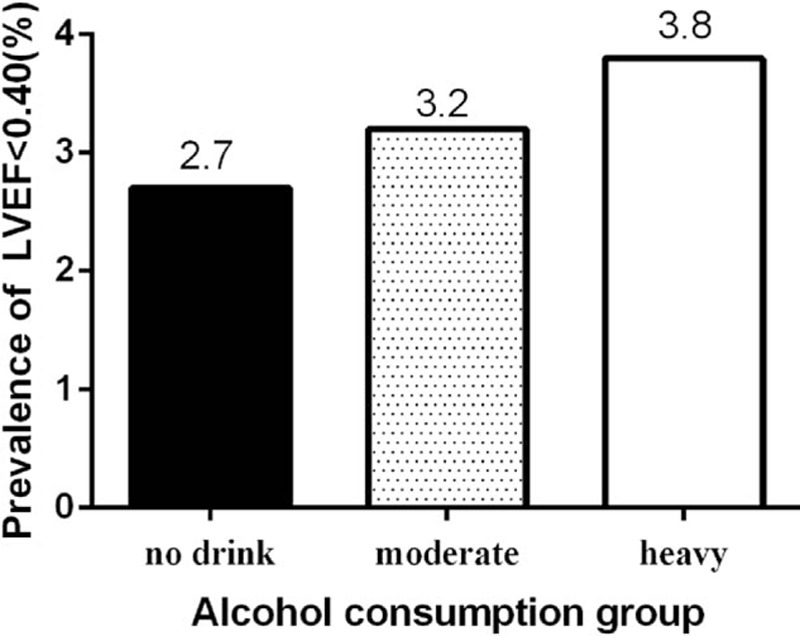
Prevalence of LVEF < 0.40 in the different alcohol consumption groups. LVEF = left ventricular ejection fraction.

As shown in Table 2, the echocardiogram results for the left ventricle in different alcohol consumption groups showed that there were no significant differences in the left ventricular internal diastolic dimensions, interventricular septal thickness, posterior wall thickness, left ventricular internal systolic dimensions, left ventricular end-diastolic volume, and left ventricular end-systolic volume among the 3 alcohol consumption groups. However, there were significant differences in the stroke volume and the minor fraction of the left ventricle among the 3 groups. The mean level of LVEF of the nondrinker, moderate, and heavy drinker groups was 0.62 ± 0.10, 0.61 ± 0.10, and 0.62 ± 0.11,respectively. Compared with the nondrinker group, the mean LVEF was lower in both the moderate and heavy drinker groups (*P* = 0.007). We used thresholds of LVEF < 0.50 and <0.40 to indicate decreased LVEF. The prevalences of LVEF <0.50 among the nondrinker, moderate, and heavy drinker groups were 10.9%, 14.6%, and 13.1%, respectively (Figure 1). The prevalences of LVEF <0.50 were higher in both the moderate and heavy drinker groups than in the nondrinker group (*P* = 0.001), and the prevalences of LVEF <0.40 among the nondrinker, moderate, and heavy drinker groups were 2.7%, 3.2%, and 3.8%, respectively (Figure 2). The prevalence of LVEF <0.40 was higher in both the moderate and heavy drinker groups than in the nondrinker group (*P* = 0.021).

**TABLE 2:**
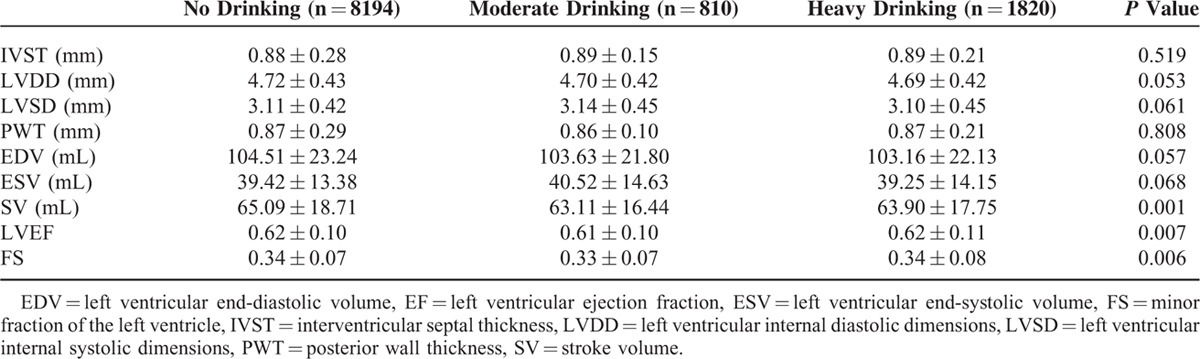
Left Ventricle Echocardiogram Results in Different Alcohol Consumption Groups

### Association Between Alcohol Consumption and LVEF

Table 3 presents multivariate logistic regression analyses of the risk of decreased LVEF corrected according to the different levels of alcohol consumption. The analyses fully adjusted for the following factors: gender, age, height, abdominal obesity, race, education, diabetes, income, current smoking, activity level, decreased GFR, history of stroke, anemia, hyperuricemia, dyslipidemia, heart rate, history of heart disease, intake of medication over the past 2 weeks, diet score, and general obesity. After these adjustments, the data showed that the moderate and heavy drinkers had an ∼1.3-fold and 1.2-fold higher risk of LVEF <0.5, respectively, than nondrinkers (OR: 1.381, 95% CI: 1.115–1.711, *P* = 0.003 for moderate drinkers; OR: 1.246, 95% CI: 1.064–1.460, *P* = 0.006 for heavy drinkers), and heavy drinkers had an ∼1.5-fold higher risk of decreased LVEF <0.4 than nondrinkers (OR: 1.482, 95% CI: 1.117–1.965, *P* = 0.006). Moderate drinkers did not show a significantly higher risk of decreased LVEF <0.4 compared with nondrinkers (OR: 1.183, 95% CI: 0.774–1.808, *P* = 0.437).

**TABLE 3:**
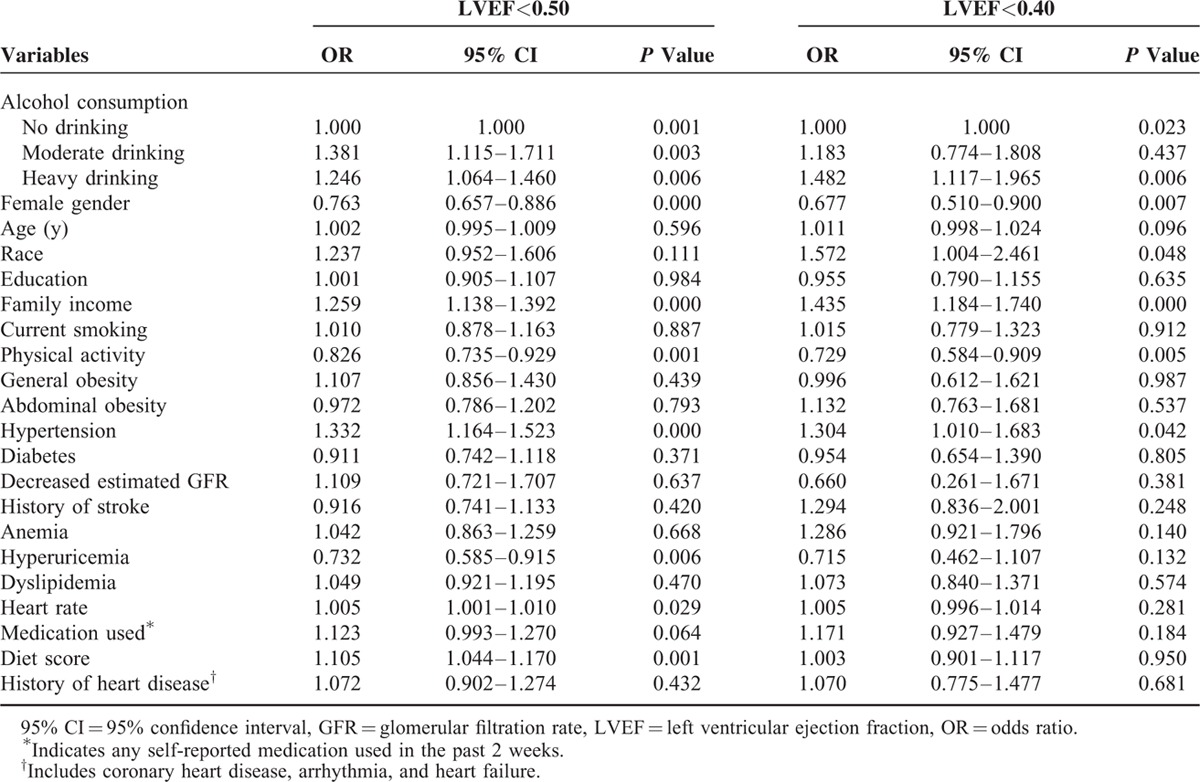
Multivariate Logistic Regression Analyses for the Association Between Increased Alcohol Consumption and Decreased Left Ventricular Ejection Fraction

## DISCUSSION

In the present study, we first evaluated the association between alcohol consumption and LVEF in a large Chinese population. We found that both moderate and heavy drinkers were at greater risk for LVEF than were nondrinkers. This is the first study to focus specifically on the relationship between alcohol consumption and LVEF in a general population in China.

Few studies have focused on the association between alcohol consumption and left ventricular ejection fraction in the general population. In a community-based, elderly population (76 ± 5 years and 60% women), higher alcohol intake was shown to be associated with lower left ventricular ejection fraction.^27^ In a population-based random sample of 2,042 adults (aged ≥45 years), a U-shaped relationship between alcohol consumption volume and LVEF was reported, and the lowest risk of moderate LV dysfunction (LVEF ≤ 40%) was observed in light drinkers (< 1 drink a day); however, no significant association was found between alcohol consumption and LV dysfunction (LVEF ≤5 0%).^28^ In the present study, both moderate and heavy alcohol consumption were associated with LVEF < 0.5. Heavy alcohol consumption was associated with both LVEF < 0.5 and LVEF < 0.4. The differences in the conclusions drawn among these studies may be due to differences in population characteristics. The present study is the first to utilize data from a rural population in China.

Based on these results, the idea that moderate alcohol consumption (≤1 drink a day for women and elderly and 1–2 drinks a day for men) is reasonable should be reconsidered.^29^ Moreover, LVEF is an important indicator of left ventricular systolic cardiac dysfunction.^30^ Our results suggest that individuals with HF should not consume moderate or excessive amounts of alcohol. However, long-term follow-up studies are needed to support this conclusion.

The mechanism of action of alcohol on the heart involves both ethanol and its metabolites, which induce reactive oxygen species generation, lipid peroxidation, inflammatory cytokine expression, organelle damage, and stress, as well as activate both the apoptotic and necrotic cell death pathways.^31–33^ For population-based studies, genetic and environmental factors should be considered. Studies have shown that the common homozygote of CHRM2 rs1824024 is significantly associated with alcohol dependence severity.^34–36^ Moreover, the echocardiogram results indicated that further studies on the relation between alcohol use and contractility of the myocardium should also be conducted.

Our study had some limitations. We analyzed the average daily alcohol consumption, but cumulative alcohol consumption was not analyzed in this study. This information will be included in the follow-up study, and the effect of cumulative alcohol consumption on LVEF could be different from the observations of this study. In addition, we did not statistically analyze whether the type of alcoholic drink consumed influenced LVEF. Several previous studies have suggested that wine intake had a relatively lower effect on cardiovascular disease.^42,43^ However, as shown in the supplementary table, nearly no wine consumption occurred within the studied population. This difference may be due to local customs and does not affect our results. In addition, our results were obtained using a cross-sectional design; thus, no cause-and-effect relationships could be established.

In conclusion, based on this general population from China, we found that increased alcohol consumption was associated with decreased LVEF compared with that of nondrinking individuals, and we recommend that the general population should consider refraining from drinking alcohol.
